# Neuroanatomical location of lung cancer brain metastases in 234 patients with a focus on cancer subtyping and biomarkers

**DOI:** 10.1371/journal.pone.0314205

**Published:** 2024-11-21

**Authors:** Michael Bonert, Jens Schittenhelm, Housne Begum, Jian-Qiang Lu, Anand Swaminath, Rosalyn A. Juergens, Alison Berzins, Jean-Claude Cutz, Asghar H. Naqvi

**Affiliations:** 1 Anatomical Pathology, Pathology and Molecular Medicine, St. Joseph’s Healthcare Hamilton, McMaster University, Hamilton, Canada; 2 Department of Neuropathology, Institute of Pathology and Neuropathology, University Hospital of Tuebingen, Eberhard Karls University of Tuebingen, Tuebingen, Germany; 3 Health Services Management, Toronto Metropolitan University, Toronto, Canada; 4 McMaster University, Hamilton, Canada; 5 Neuropathology, Pathology and Molecular Medicine, Hamilton Health Sciences, McMaster University, Hamilton, Canada; 6 Radiation Oncology, Oncology, Hamilton Health Sciences, McMaster University, Hamilton, Canada; 7 Medical Oncology, Oncology, Hamilton Health Sciences, McMaster University, Hamilton, Canada; Showa University Fujigaoka Hospital, JAPAN

## Abstract

Brain metastases are frequent in neuropathology practices; however, the literature on their distribution is frequently derived from imaging studies. This work examined metastases of lung cancer to the brain through the lens of pathology specimens. All brain surgical pathology cases accessioned from 2011–2020 were retrieved from a regional laboratory. Specimens were classified by neuroanatomical location, diagnostic category, and diagnosis with a hierarchical free text string-matching algorithm. All reports classified as probable metastasis per algorithm were reviewed by a pathologist. Lung biomarkers and selected immunostains were retrieved with text parsing and reviewed. Among 4,625 cases of brain surgical resection specimens, 854 were classified as probable metastasis by the algorithm. On report review, 538/854 cases were confirmed as metastasis with a known primary site. The 538 cases were from 511 patients and 234/511 patients had lung primaries. Small cell lung cancer lesions were most frequently found in the cerebellum (17/30). Lesions from lung adenocarcinoma (59/164) and non-small cell carcinoma-not otherwise specified (NSCLC-NOS) (15/34) were most commonly found in the frontal lobe. Squamous cell carcinoma lesions were most commonly found in the frontal and occipital lobes (8/27). 72/234 cases were reported as NSCLC-NOS and could be further subclassified using immunostaining (41/72). Lung biomarker data were retrieved in ~38% of cases. PD-L1 positivity was dependent on neuroanatomical distribution (p = 0.04); other examined biomarkers were not. The distribution of lung tumours metastatic to the brain is dependent on the lung cancer subtype (p<0.001). The reporting of histologic subtype could be further optimized in the local environment.

## Introduction

Lung cancer is the most common cause of brain metastasis (BM) and the incidence of lung cancer BM has risen in the last decades due to longer survival of patients, and increased utilization of imaging of the brain during treatment follow-up [1]. This has been accompanied by a year-over-year decrease in lung cancer BM mortality that is approaching a plateau [2].

Among lung cancers, adenocarcinoma and small cell carcinoma are the most common histological diagnoses and the incidence of non-small cell lung cancer correlates with patient age and advanced tumour stage [3]. BM is detected in 21% of patients with lung cancer at the time of diagnosis; in stage IV disease, the CNS is the first site to manifest metastatic disease in 35% of patients [4].

In these specimens, the pathologist is typically called upon to (a) confirm that it is a lung metastasis, (b) histologically type it (typically using morphology and immunostains), and (c) biomarker testing (e.g. EGFR, ALK, ROS-1, NTRK, KRAS, PD-L1, MET exon 14 skipping, etc), required for patient-centered oncology. The location of brain metastases is known to depend on the primary site [5,6]. We have previously shown that the distribution of histologically verified metastatic tumours in the brain is similar to data predicted by the radiological evaluation [7]. Wang *et al*. [5] examined 335 lung cancer patients and found (based on imaging) that lung adenocarcinomas metastasize predominantly to the frontal lobe, while small cell carcinomas (SCLC) and squamous cell carcinomas show predilection for the cerebellum [5,6].

Capper *et al*. [8] examined brain metastases for mutations and among their lung cancer cohort, 83% were non-small cell lung cancer (NSCLC) and 16% were SCLC [8]. Among the NSCLC cohort, 47% were diagnosed as adenocarcinoma, 9% as squamous cell carcinoma, 9% as large cell carcinoma and 2% as adenosquamous carcinoma while 14% were classified as NOS. Demleitner *et al*. [9] reviewed their surgical records and found that lung cancer BM’s consisted of 64% adenocarcinomas, 16% squamous cell, 10.5% large cell, 0.6% adenosquamous and 1% NOS carcinomas [9]. The most common immunostains used in lung cancer BM workups are TTF-1, cytokeratin 7 (CK7) and p63. Approximately 81% of all NSCLCs are TTF-1 positive [9] and up to 98% are CK7 positive [10].

Relevant molecular genetic alterations such as EGFR and KRAS mutations, ALK, ROS-1, RET or NTRK fusions are detected in less than half of patients [11]. A review by Ali *et al*. [12] indicated that tumour molecular heterogeneity (characterized by discordant results for a BM and primary) may be present in greater than 15% of patients.

Most lung cancer BM neuroanatomic distribution data is centered on brain imaging studies and focuses on the immunohistochemistry and molecular results obtained from the primary tumour. In this study, building on our prior work on brain metastases [7], we aimed to identify and type a lung cancer BM cohort retrospectively by employing a diagnostic algorithm on pathology reports of metastatic tumors.

## Methods

Ethics approval was obtained to review the pathology of patients with at least one surgical neuropathology specimen (Hamilton Integrated Research Ethics Board (HiREB) 14453-C). The need for patient consent was waived by the ethics board, due to the study design.

On May 12, 2022, all in-house brain surgical pathology cases accessioned January 1, 2011 to December 31, 2020, were retrieved for analysis. Following data retrieval, the data was anonymized. Patients in the data set could not be identified by the authors after anonymization.

The project builds off a prior project in which, specimens were classified by anatomical location, diagnosis, and diagnostic category with a hierarchical free text string-matching algorithm (HFTSMA) [7]. The diagnostic classification of the HFTSMA was audited via (a) 200 randomly selected cases, and (b) 200 random cases categorized as probable metastases.

The project also draws on a study which reviewed reports of lung core biopsies for neoplastic disease (ethics approval HiREB 3811) presented at the *United States and Canadian Academy of Pathology* Annual Meeting in Los Angeles, USA (2020), and on knowledge acquired parsing free-text pathology reports [13,14].

Diagnoses were grouped into six mutually exclusive groupings in a hierarchy (unclassified, brain cardiovascular disease, miscellaneous lesions, other brain lesions, probable metastasis, primary brain tumour); details are within our prior study [7].

All reports classified as probable metastasis with one primary site by the computer were reviewed by a pathologist. In patients with several pathology cases with metastatic disease, the first case was retained and the remaining cases removed from the data set. Lung biomarker data was extracted from the pathology reports in a separate process and then merged with the output of the HFTSMA. Captured lung biomarkers included EGFR, ALK by immunostaining, ALK by fluorescence in situ hybridization, PD-L1, RAS, BRAF V600 and ROS-1. All the neuroanatomical locations of the lung brain metastases were reviewed by a pathologist to ensure accuracy.

Selected immunostain results were extracted with a logical text parsing tool. The TTF-1 and p63 results for lung cases in the study set were reviewed and (the logical text parsing tool results) were corrected where necessary.

**Fig 1:**
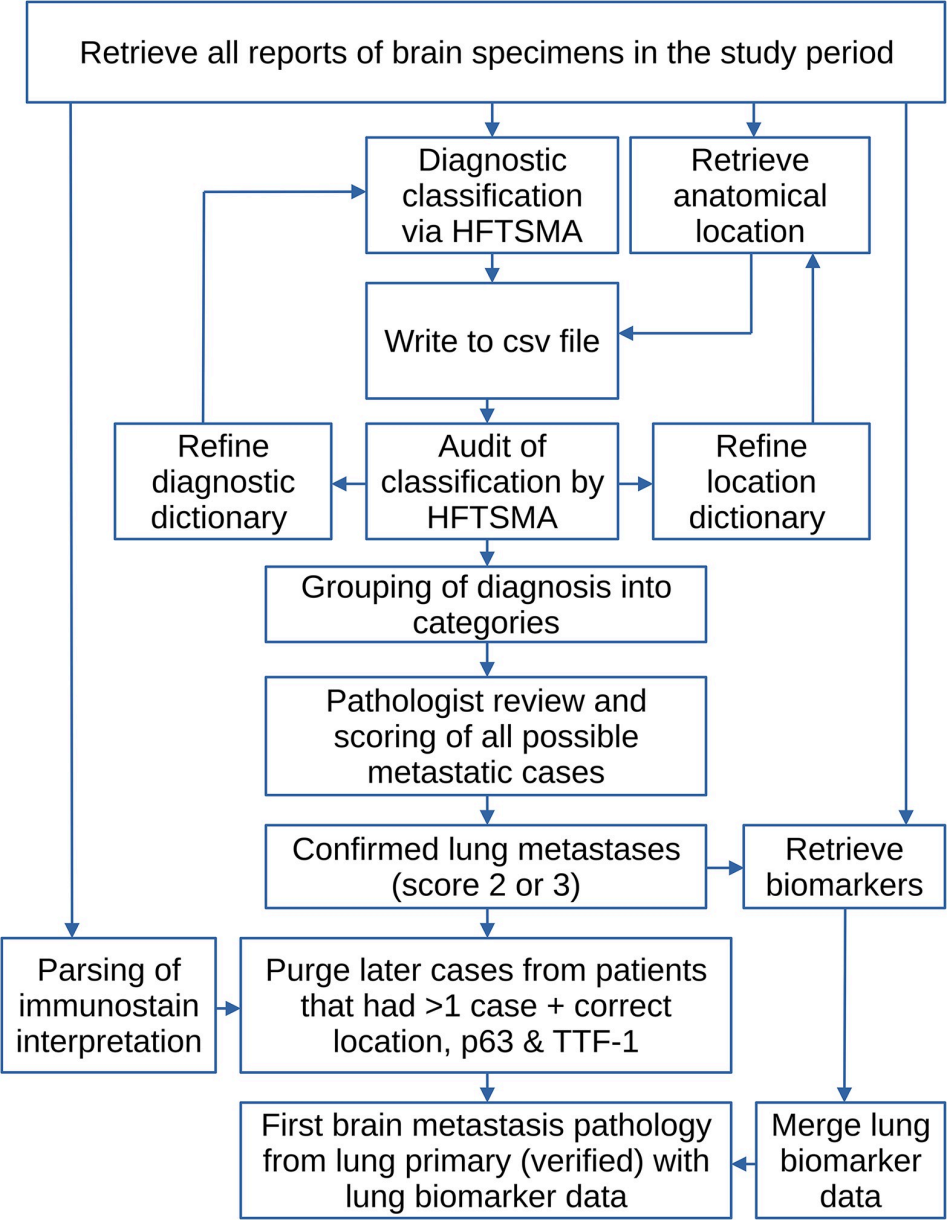
The study methods in a flow chart. HFTSMA = hierarchical free text string-matching algorithm; csv = formatted text output (tab or comma separated).

The methods are summarized in a flow chart—see Fig 1.

NSCLC cases that were TTF-1 positive *and* p63 negative were re-classified as *adenocarcinoma*. NSCLC cases that were TTF-1 negative *and* p63 positive were re-classified as *squamous cell carcinoma*. NSCLC cases that did not meet either criteria for re-classification were deemed to be *non-small cell carcinoma-not otherwise specified* (NSCLC-NOS).

Tabulation was done using LibreOffice Calc (https://www.libreoffice.org/). Statistical testing was done using a script written in R (https://search.r-project.org). Fisher’s exact test was done using the R base function (https://search.r-project.org/CRAN/refmans/rstatix/html/fisher_test.html). Clustered heatmaps were created using the *Pretty Heatmaps* package (https://cran.r-project.org/web/packages/pheatmap/index.html)

## Results

The time frame had 4,625 brain surgical pathology cases. As noted in our prior study, the HFTSMA could classify 4,556 of 4,625 cases from a diagnostic perspective and 4,577 of 4,625 from a neuroanatomical location perspective [7].

The HFTSMA identified 854 probable metastases and a pathologist reviewed all of the pathology reports from those cases. Cases were scored 0 to 3, as described in our prior paper [7]. In total, 538 of 854 cases were scored 2 or 3; these 538 cases were from 511 patients.

The location classification was correct in 94% (480/511) of the verified cases. More details on the case classification are found in S10 and S11 Tables in S3 File.

234 of 511 patients had a lung primary tumour. One hundred cases (43%) classified as lung metastases had a second report review by a pulmonary pathologist.

### Patient with lung primaries and multiple specimens

Eight patients with a lung primary had more than one BM pathology specimen in the study time period; all eight had two specimens. In six of eight patients the anatomical site of the first and subsequent specimens matched.

### First brain pathology with metastatic disease from the lung

The remaining analysis is focused on the first brain pathology specimen with metastatic disease from the lung in the study time frame.

The neuroanatomical location could be determined in 233 of 234 patients; in one patient the location of the lesion was unknown. In 36 patients, the location codes overlapped/multiple locations were given. The overlap resulted from cases that included several keywords, e.g. ‘frontal’ and ‘temporal’ or multiple parts. The occurrence of overlapping sites is shown in Table 1.

**Table 1:** Neuroanatomical site overlap (occurrence of multiple lesions/lesions involving multiple neuroanatomical sites).

Site	Frontal	Parietal	Temporal	Occipital	Cerebellum	Other	Unknown	Misc.
Frontal	88	4	4	0	0	1	0	-
Parietal	4	48	2	9	0	2	0	-
Temporal	4	2	20	0	0	0	0	-
Occipital	0	9	0	35	2	2	0	-
Cerebellum	0	0	0	2	61	10	0	-
Other	1	2	0	2	10	17	0	-
Unknown	0	0	0	0	0	0	1	-
Sum	97	65	26	48	73	32	1	234
Diagonal	88	48	20	35	61	17	1	-
Off-diagonal	9	17	6	13	12	15	0	-
Pure	79	31	14	22	49	2	1	198
Mixed	-	-	-	-	-	-	-	36

Misc. = Miscellaneous.

## Classification by the diagnosis in the pathology report

A preliminary analysis considered the number of patients, specimens by the diagnosis and the neuroanatomical location. The results were tabulated (S1, S2a and S3a Tables in S3 File) and plotted (S1 and S2 Figs), and it was noted that: non-small cell carcinoma was highly prevalent (S4, S5 Tables in S3 File). This prompted a further analysis and a refinement using the reported immunohistochemistry findings.

Immunostains TTF-1 and p63 were done on 206 and 46 of 234 (lung cancer) patients respectively. Immunostaining for p40 was done in only five lung cancer cases; this stain was not included in the analysis. The classification by immunohistochemical staining was tabulated (S6 Table in S3 File) and used to refine the diagnostic classification (S7 Table in S3 File).

The classification of SCLC was based on the reported classification and in 25 of 28 cases supported by immunohistochemistry that included at least one neuroendocrine marker.

### Lung cancer histologic subtype and immunohistochemical staining

The number of patients and (metastatic) lesions (specimens) by immunostain-refined lung subtype and neuroanatomical location is shown in the Table 2. Individual patients can have lesions at different neuroanatomical sites and/or lesions that involve multiple neuroanatomical sites; thus, the number of lesions exceeds the number of patients.

**Table 2:** Lung cancer subclassification using immunostaining by patients and lesions.

Lung Cancer Type	Frontal	Parietal	Temporal	Occipital	Cerebellum	Unknown /Other	Lesions	Patients
Adenocarcinoma *	59	31	14	21	37	2	164	152
Squamous cell carcinoma **	8	5	5	8	1	0	27	23
Small cell carcinoma	6	4	0	2	17	1	30	28
Non-small cell carcinoma NOS ***	15	8	1	4	6	0	34	31
Sum	88	48	20	35	61	3	255	234

* Adenocarcinoma definition: TTF-1 positive, p63 NOT positive.

** Squamous cell carcinoma definition: TTF-1 negative, p63 positive.

*** Includes probable Large Cell Neuroendocrine Carcinoma (LCNEC).

**Fig 2:**
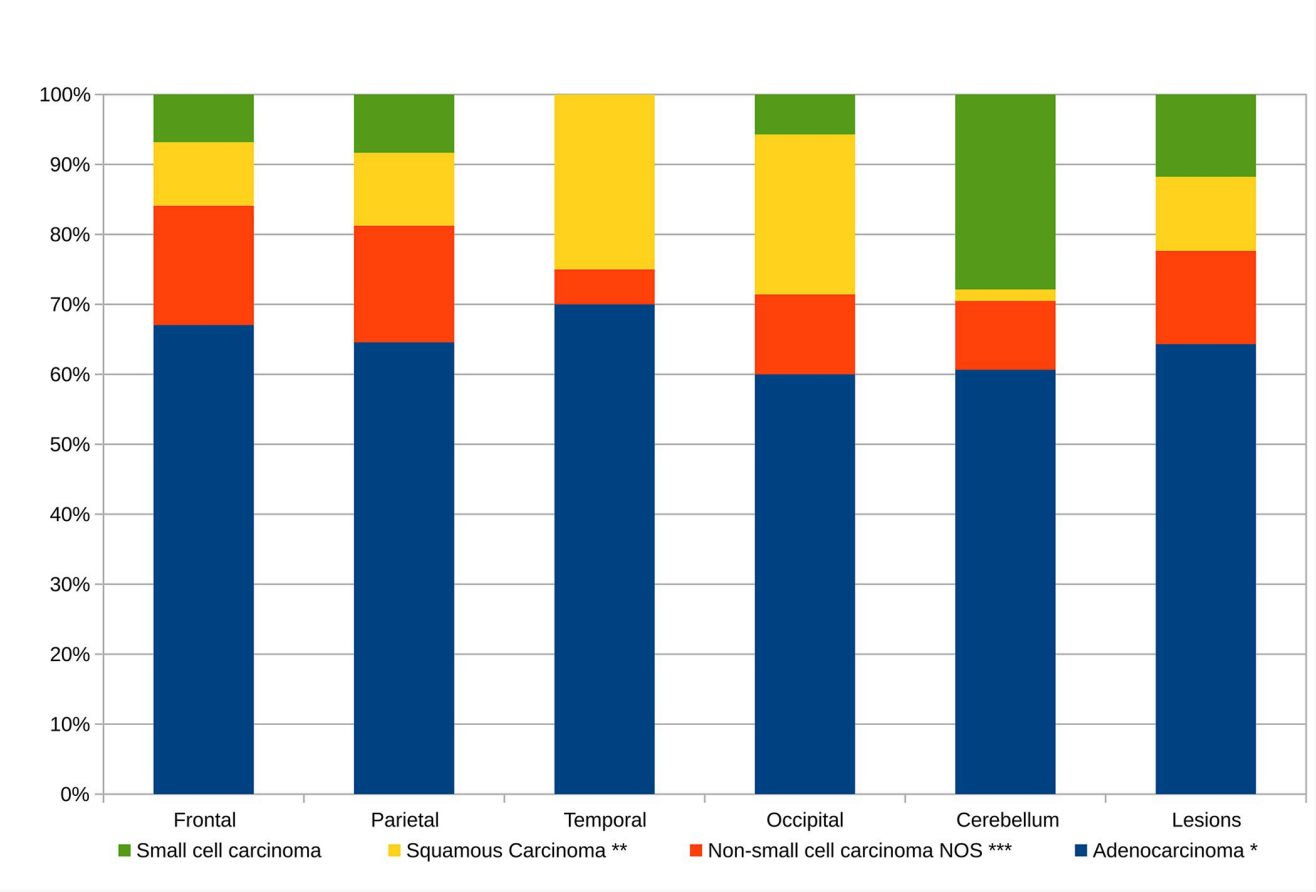
The brain metastases from the lung by neuroanatomical site. This figure is applicable in the diagnostic context; if the neuroanatomical site is known, it answers the question “How likely is a metastasis from a lung tumour of subtype ‘X’?”.

The immunostain-refined pathologic classification by neuroanatomical site (calculated from Table 2) is given in S2b Table in S3 File and Fig 2; it mirrors probabilities the pathologist has of making a specific subtype diagnosis when given the neuroanatomical site. Adenocarcinoma is the most prevalent in all neuroanatomical sites.

**Fig 3:**
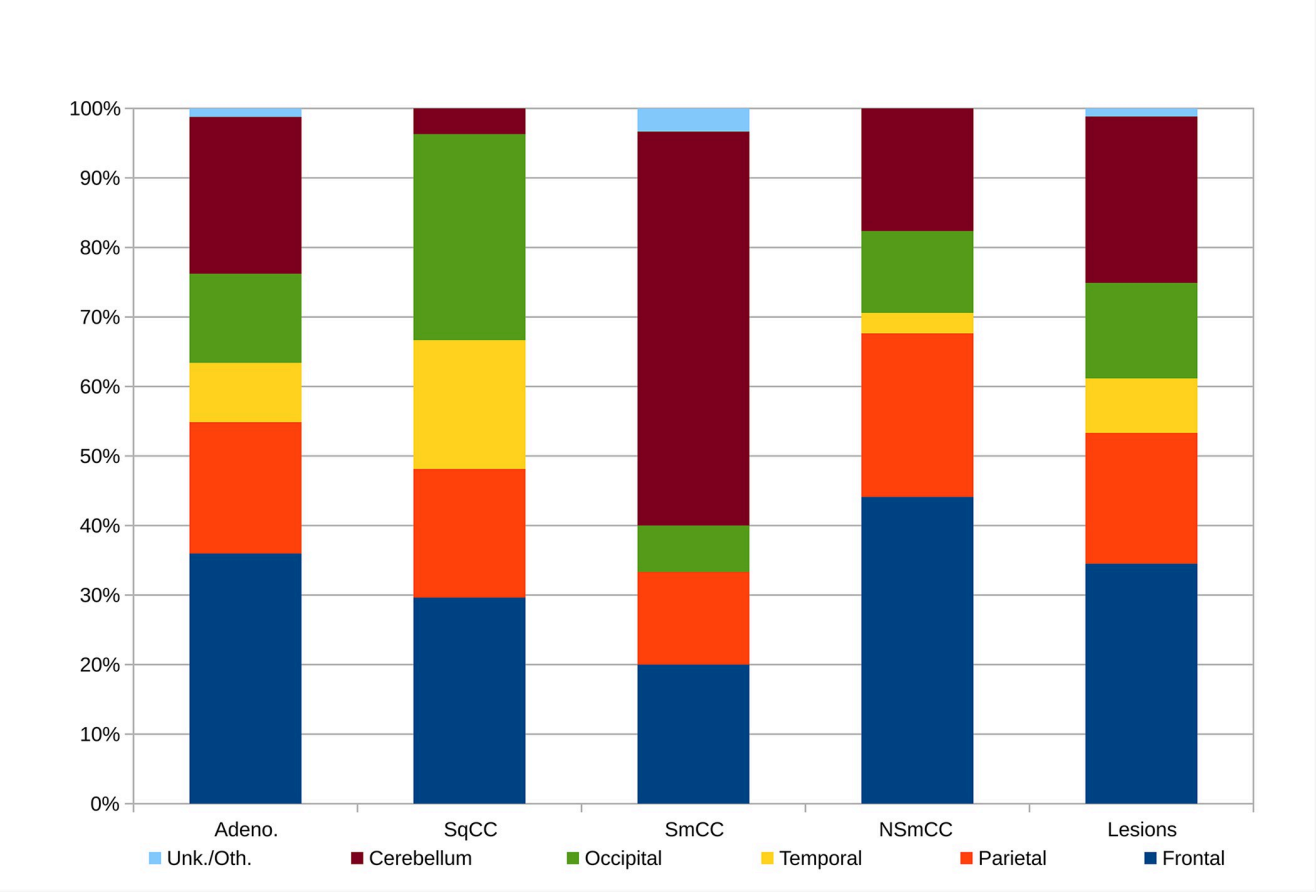
The brain metastases by lung subtype. This figure is applicable in the pathobiologic context; if the lung subtype is known, it answers the question “How likely is a metastasis to the neuroanatomical site ‘Y’?”.

The distribution of the immunostain-refined lung subtypes (calculated from Table 2) is given in S3b Table in S3 File and Fig 3; it mirrors the probabilities of a given subtype being found at a specific neuroanatomical site.

Small cell lung cancer was most frequently found in the cerebellum. Lung adenocarcinoma, and lung non-small cell carcinoma-not otherwise specified were mostly commonly found in the frontal lobe specimens. Squamous cell carcinoma was most commonly found in the frontal and occipital lobe specimens.

The distribution of adenocarcinoma and non-small cell carcinoma-not otherwise specified (NOS) are similar.

### Statistical analysis for neuroanatomical location and histologic subtype

**Fig 4:**
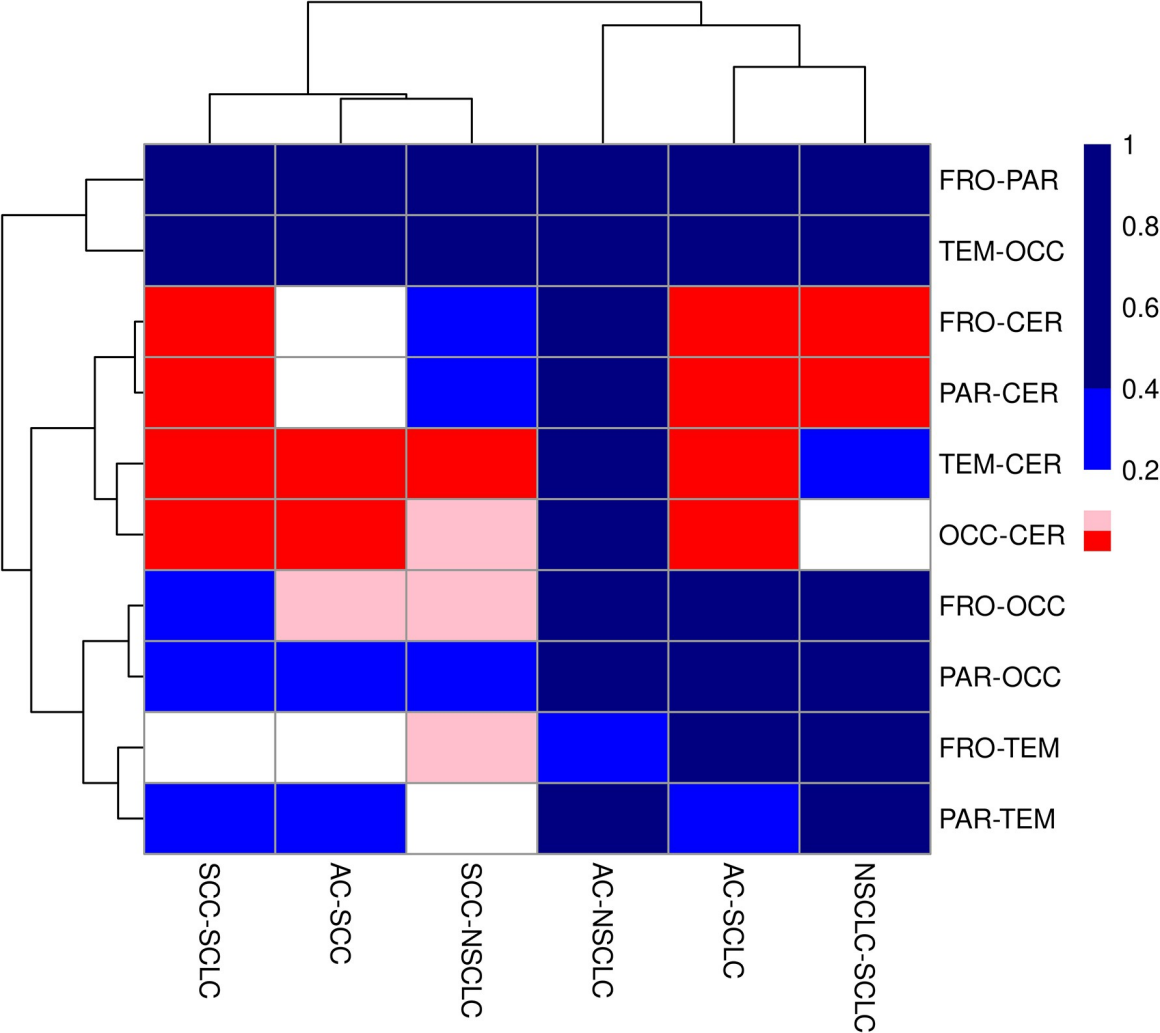
Clustered heatmap showing the p-values calculated by Fisher’s exact test for all each pairing of neuroanatomical location and lung cancer subtype. Neuroanatomical location abbreviations: FRO = frontal lobe, PAR = parietal lobe, TEM = temporal lobe, OCC = occipital lobe, CER = cerebellum. Lung cancer subtype abbreviations: AC = adenocarcinoma, SCC = squamous cell carcinoma, SCLC = small cell carcinoma, NSCLC = non-small cell carcinoma-NOS. The colours represent different p-value ranges: red is p = 0 to <0.05, pink is p = 0.05 to <0.1, white is p = 0.1 to <0.2, light blue is p = 0.2 to <0.4 and dark blue is p = 0.4 to 1.0.

The neuroanatomical location versus the lung cancer subtype was formally evaluated using Fisher’s exact test (FET) after simplifying Table 1 - by removing the one case that had only had ‘unknown/other’ for the neuroanatomical location. The resulting (simplified) contingency table had 252 lesions in 20 cells; a FET demonstrated statistically significant differences between the cells (p = 0.00067). To further evaluate the interaction between lung cancer subtype and neuroanatomical location, all possible 2x2 contingency tables (n = 60) were constructed. P-values were calculated for each pairing of neuroanatomical location and lung cancer subtype using FET and results visualized using a clustered heatmap—see Fig 4.

The heatmap demonstrates statistical differences are between the cerebellum and other neuroanatomical sites. A lack of statistical differences (p>0.2) were seen for comparisons between (1) adenocarcinoma and non-small cell carcinoma-not otherwise specified, (2) frontal lobe and parietal lobe, (3) temporal lobe and occipital lobe, and (4) parietal lobe and occipital lobe.

To assess the impact of the 36 patients with overlapped location codes/multiple locations, the data was re-calculated without those 36 patients. With those 36 patients removed and unknown/other cases purged, the data set consisted of 195 patients with one lesion and a contingency table with 20 cells; the Fisher’s exact test for neuroanatomical location (frontal, parietal, temporal, occipital, cerebellum) versus lung subtype (AC, SCC, SCLC, NSCLC) was likewise significant (p = 0.00453). A heatmap was also generated (for the set of 195 patients) from all possible 2x2 contingency tables (n = 60)—see S3 Fig; it reproduced the main trends seen in Fig 4.

### Lung biomarker findings

Ninety cases included lung biomarker reporting of some kind. An overview of the available biomarkers testing is seen in Table 3.

**Table 3:** Selected lung biomarkers.

Biomarker	Adequate	Negative	Low Positive	Positive	Percent Negative	Percent Low Pos.	Percent Positive
**EGFR**	65	57	NA	8	87.7%	NA	12.3%
**ALK**	78	77	NA	1	98.7%	NA	1.3%
**PD-L1**	79	27 †	15 *	37 **	34.2% †	19.0% *	46.8% **
**RAS**	51	28	NA	23	54.9%	NA	45.1%
**BRAF V600**	51	50	NA	1	98.0%	NA	2.0%

NA = not applicable.

**†** = <1% tumour proportion score (TPS).

* = 1 to 49% TPS.

** 50% or more TPS.

No biomarker data could be retrieved for the time period 2011–2013. In the period 2016–2020, 125 patients with metastatic lung cancer were diagnosed. 87 of the 125 (69.6%) had biomarker testing of some kind.

Selected biomarkers (EGFR, PD-L1, RAS) were stratified by reported lung cancer subtype in Table 4 (IHC modified) / S8 Table (unmodified) in S3 File and neuroanatomical location in Table 5.

**Table 4:** Biomarkers and cancer subtype.

Biomarker and Status	AC	SCC	NSCLC***	SCLC	Sum
EGFR					
Adequate	48	7	9	1	65
Negative	43	7	6	1	57
Positive	5	0	3	0	8
PD-L1					
Adequate	60	7	11	1	79
Negative	19	3	4	1	27
Low positive	12	1	2	0	15
Positive	29	3	5	0	37
RAS					
Adequate	36	6	8	1	51
Negative	16	5	6	1	28
Positive	20	1	2	0	23
Subtype fraction	0.650	0.098	0.132	0.120	1.000
Number of cases	152	23	31	28	234

AC = adenocarcinoma; SCC = squamous cell carcinoma; SCLC = small cell carcinoma.

*** Includes probable Large Cell Neuroendocrine Carcinoma (LCNEC).

**Table 5:** Biomarkers and neuroanatomical sites.

Biomarker and Status	Frontal	Parietal	Temporal	Occipital	Cerebellum	Sum
EGFR						
Adequate	27	17	4	12	11	71
Negative	22	16	4	12	9	63
Positive	5	1	0	0	2	8
PD-L1						
Adequate	27	25	5	17	13	87
Negative	6^‡^^✣^	9^‡^	5^‡^^‡^^ß^	5^ß^^ß^	6^✣^	31
Low positive	4	3	0	5	4	16
Positive	17^‡^^‡^^✣^	13^‡^	0^‡^^‡^^ß^	7^ß^^ß^	3^✣^	40
RAS						
Adequate	21	12	3	12	8	56
Negative	12	8	2	8	3	33
Positive	9	4	1	4	5	23
Site fraction	0.349	0.190	0.079	0.139	0.242	1.000
Neuroanatomical site **	88	48	20	35	61	252

** Three specimens are unknown / other location; these are not included in this table.

† p<0.05 for 2x2 comparison of Parietal-Temporal versus PD-L1 negative-PDL1 positive.

‡ p<0.05 for 2x2 comparison of Frontal-Temporal versus PD-L1 negative-PDL1 positive.

ß p<0.05 for 2x2 comparison of Temporal-Occipital versus PD-L1 negative-PDL1 positive.

✣ p<0.05 for 2x2 comparison of Frontal-Cerebellum versus PD-L1 negative-PDL1 positive.

### Statistical analysis of biomarker data

Fisher’s exact tests were done to assess the relationship between EGFR and subtype (excluding small cell carcinoma), PD-L1 and subtype (excluding small cell carcinoma), and RAS and subtype (excluding small cell carcinoma); none of the tests reached statistical significance (p>0.05).

Fisher’s exact tests were done individually for EGFR and neuroanatomical location, PD-L1 and neuroanatomical location, and, RAS and neuroanatomical location. Only, PD-L1 was predicted by neuroanatomical location (p = 0.03654). In 2x2 comparisons for PD-L1 (PD-L1 negative/PD-L1 low positive/PD-L1 positive) and neuroanatomical location (frontal lobe/parietal lobe/temporal lobe/occipital lobe/cerebellum), only comparisons between PD-L1 negative and PD-L1 positive reached statistical significance (p<0.05); statistically significant differences were present between (1) parietal lobe and temporal lobe, (2) frontal lobe and temporal lobe, (3) temporal lobe and occipital lobe, and (4) frontal lobe and cerebellum–as shown in S4 Fig.

## Discussion

The project built on our prior successful extraction of information from partially structured free text reports.

The aggregated data from the reports appear to be useful to understand patterns of disease spread and corresponds to the spread pattern seen radiologically. The findings may be useful to improve understanding of metastatic disease, especially with the decline in hospital autopsy rates. The tumour brain microenvironment (TBME) in metastatic cancer is emerging as a research area [15,16]; this work raises questions about how cancer subtype, neuroanatomical location and TBME may interact. Several such questions are: *Do different lung cancer subtypes at the same neuroanatomical location share common molecular changes*? *Can brain gene expression profiles by neuroanatomical location* (such as by *Negi and Guda* [17]) *yield additional insights when compared to tumour expression profiles by neuroanatomical site*?

This data set represents a limited perspective into metastatic lung cancer to the brain, as many metastatic brain lesions are not excised or biopsied. Also, biopsies of the brain are typically taken from easy to access non-critical neuroanatomical locations.

Brain metastases that are biopsied are typically seen in one of the following scenarios: (a) prove that a candidate primary lesion (seen on cross-sectional imaging) is the source of the lesion in the brain, (b) radiologically suspected metastasis and unknown primary, (c) prove the recurrence of a prior known cancer and, if the patient had several primaries, determine which cancer has spread to brain. As noted in our prior work, these scenarios represent a specific time point in the progression of a cancer [7]. As such, they are also reflective of the lung cancer biology to some extent.

### Neuroanatomical location

As in our prior work on brain metastases, cases reported to involve multiple neuroanatomical locations complicated the analysis. As the number of cases with multiple neuroanatomical location codes (36/234 or 15.4%) were a small subset of cases, an initial simple analysis with one neuroanatomical site per patient reproduced the same trends for the distribution of lung cancer subtypes.

Lesions present in several different neuroanatomical locations could represent a slightly different disease (multiple brain metastases versus singular brain metastasis).

Cases with several specimens sampling the same neuroanatomical location, e.g. ‘temporal’ most likely represent two samples from the same lesion or possibly two samples from different lesions in the same neuroanatomical site (e.g. ‘temporal lesion #1’ and ‘temporal lesion #2’). Samples taken from two neighbouring lobes (e.g. ‘temporo-parietal’) may originate from two different lesions in different lobes or one large lesion. It is not possible to determine which scenario applies based on the information included in the report.

### Lung cancer subtypes

Wang *et al*. found that small cell carcinoma and squamous cell carcinoma preferentially metastasize to the cerebellum, and adenocarcinoma to the frontal lobe [5]. This study shows concordance with respect to adenocarcinoma and small cell carcinoma, but not squamous cell carcinoma.

The reporting of lung cancer subtype varied significantly from year to year. The underlying cause of this cannot be ascertained from the study. The authors suspect that it may be driven by changes in reporting guidelines over the study time period.

It should be noted that in the HFTSMA hierarchy ‘adenocarcinoma’ and ‘squamous cell carcinoma’ both supercede ‘non-small cell carcinoma’. For example, cases signed out as ‘non-small cell carcinoma, favour adenocarcinoma’ would be classified as ‘adenocarcinoma’.

Subclassification of non-small cell carcinoma is considered critical from an oncologic perspective [18]. It can usually be done with immunostaining for p63 (or preferably p40) and TTF-1 [19].

The local lung core biopsy cohort signed out by surgical pathologists showed a non-small cell carcinoma-not otherwise specified reporting rate of approximately 10% (unpublished data); this differed significantly from the local brain biopsy cohort reported by neuropathologists, where 72/234 patients (31%) were classified as non-small cell carcinoma-not otherwise specified.

The non-small cell carcinoma cases in this cohort could be further analyzed with the reported immunostains (S6 and S7 Tables in S3 File). Removal of the 37 cases consistent with adenocarcinoma (definition: TTF-1 positive and p63 NOT positive) and 4 cases consistent with squamous cell carcinoma (definition: TTF-1 negative and p63 positive) did not result in a significant shift of disease distribution (compare S2a Table in S3 File with S2b and S3a in S3 File with S3b Table in S3 File); a nearly identical fraction of lesions in the (revised) non-small cell carcinoma grouping were in the frontal lobe (15/34 or 44% revised with IHC versus 35/78 or 45% unadjusted) and cerebellum (6/34 or 18% revised with IHC versus 13/78 or 17% unadjusted).

If tissue is adequate, we believe the barest minimum should be that: TTF-1 and p40 is done on suspected lung metastases to the brain, if a non-small cell carcinoma subtype has not been determined on prior pathology. Ao *et al*. advocate for the use of TTF-1, p40 and napsin A [20]. We believe this is a reasonable standard; napsin A is a useful addition as it is almost never positive in small cell carcinoma [21]. In the context of reporting language, we recommend that guidelines given by the International Association for the Study of Lung Cancer/American Thoracic Society/European Respiratory Society Classification for small biopsies should be followed by neuropathologists [22].

### Lung biomarkers

Findings in this cohort are largely inconclusive within the context of named neuroanatomical sites. Since EGFR mutation status was reported to be predicted by the depth of metastatic deposits in the brain [23], we suspected that neuroanatomical location difference might be seen. It is possible that our cohort is underpowered to detect such differences.

The significant differences seen in PD-L1 status by neuroanatomical location suggest that the temporal lobe and cerebellum may represent unique neuroanatomical micro-environments.

It should be noted that the standard in breast cancer pathology is to retest (recurrent/relapsed) breast cancer surgical pathology specimens for biomarkers [24]. It is apparent that this same standard is not practised for lung cancer metastatic to the brain. As lung BMs may show different biomarker profiles from the primary lung lesion, it may be prudent to institute reflex biomarker re-testing, especially in pre-treated patients or patients with any clinicopathological discordance.

## Conclusion

This study represents a unique window into the biology of metastatic lung cancer to the brain, as pathologists see a unique subset of brain metastases specimens. The ordering of lung biomarkers and reporting of cancer subtype could be optimized in our environment. The distribution of the metastatic lung cancer in the brain is dependent on the lung cancer subtype (p<0.001). PD-L1 status is predicted by the neuroanatomical location (p<0.04). Future work should correlate the pathologic findings with radiologic findings to obtain a more complete picture and examine the molecular changes in lung cancer subtypes found at different neuroanatomical locations.

## Supporting information

S1 File Data file description. (DOCX)

S2 File Anonymized data. (XLS)

S3 File All tables and figures. (XLSX)

S1 Fig Lung subtype by neuroanatomical site (S1 Table in S3 File divided by site—diagnostic perspective)—reported subtype. (TIF)

S2 Fig Distribution by lung cancer subtypes (S1 Table in S3 File divided by subtype—pathobiologic perspective)—reported subtype. (TIF)

S3 Fig Fisher’s exact test P-Values for cases with one lesion only. (TIF)

S4 Fig Fisher’s exact test P-Values for cases with one or more lesions and PD-L1 status. (TIF)

## References

[1] **<**References>. Preusser M, Capper D, Ilhan-Mutlu A, Berghoff AS, Birner P, Bartsch R, et al. Brain metastases: pathobiology and emerging targeted therapies. Acta Neuropathol. 2012;123(2):205–22. https://pubmed.ncbi.nlm.nih.gov/22212630/. doi: 10.1007/s00401-011-0933-9 22212630

[2] Che W, Liu J, Fu T, Wang X, Lyu J. Recent Trends in Synchronous Brain Metastasis Incidence and Mortality in the United States: Ten-Year Multicenter Experience. Curr Oncol. 2022;29(11):8374–89. https://pubmed.ncbi.nlm.nih.gov/36354720/. doi: 10.3390/curroncol29110660 36354720 PMC9689090

[3] Schouten LJ, Rutten J, Huveneers HA, Twijnstra A. Incidence of brain metastases in a cohort of patients with carcinoma of the breast, colon, kidney, and lung and melanoma. Cancer. 2002;94(10):2698–705. https://pubmed.ncbi.nlm.nih.gov/12173339/. doi: 10.1002/cncr.10541 12173339

[4] Merkin RD, Chiang VL, Goldberg SB. Management of patients with brain metastases from NSCLC without a genetic driver alteration: upfront radiotherapy or immunotherapy? Therapeutic Advances in Medical Oncology. 2023;15:17588359231175438. https://www.ncbi.nlm.nih.gov/pmc/articles/PMC10233588/. doi: 10.1177/17588359231175438 37275964 PMC10233588

[5] Wang G, Xu J, Qi Y, Xiu J, Li R, Han M. Distribution Of Brain Metastasis From Lung Cancer. Cancer Manag Res. 2019;11:9331–8. https://www.ncbi.nlm.nih.gov/pmc/articles/PMC6830371/. doi: 10.2147/CMAR.S222920 31802951 PMC6830371

[6] Cardinal T, Pangal D, Strickland BA, Newton P, Mahmoodifar S, Mason J, et al. Anatomical and topographical variations in the distribution of brain metastases based on primary cancer origin and molecular subtypes: a systematic review. Neurooncol Adv. 2022;4(1):vdab170. https://pubmed.ncbi.nlm.nih.gov/35024611/. doi: 10.1093/noajnl/vdab170 35024611 PMC8739649

[7] Bonert M, Berzins A, Begum H, Schittenhelm J, Lu JQ, Juergens RA, et al. Neuroanatomical location of brain metastases from solid tumours based on pathology: An analysis of 511 patients with a comparison to the provided clinical history. PLoS One. 2023 Nov 9;18(11):e0294154. https://pubmed.ncbi.nlm.nih.gov/37943775/#full-view-affiliation-2. doi: 10.1371/journal.pone.0294154 37943775 PMC10635465

[8] Capper D, Berghoff AS, Magerle M, Ilhan A, Wöhrer A, Hackl M, et al. Immunohistochemical testing of BRAF V600E status in 1,120 tumor tissue samples of patients with brain metastases. Acta Neuropathol. 2012;123(2):223–33. https://pubmed.ncbi.nlm.nih.gov/22012135/. doi: 10.1007/s00401-011-0887-y 22012135

[9] Demleitner M, Erlenbach-Wünsch K, Coras R, Erber R, Polifka I, Eyüpoğlu I, et al. Lung cancer presenting with central nervous system metastasis: Clinicopathological and molecular analysis of 171 cases. Ann Diagn Pathol. 2023;63:152082. https://pubmed.ncbi.nlm.nih.gov/36634550/. doi: 10.1016/j.anndiagpath.2022.152082 36634550

[10] Fernandez C, Liprandi A, Bouvier-Labit C, Figarella-Branger D. [Value of cytokeratin 7 and 20 for the diagnosis of cerebral metastases of adenocarcinoma: study of 78 cases]. Ann Pathol. 2001;21(2):129–35. https://pubmed.ncbi.nlm.nih.gov/11373582/. 11373582

[11] Villalva C, Duranton-Tanneur V, Guilloteau K, Burel-Vandenbos F, Wager M, Doyen J, et al. EGFR, KRAS, BRAF, and HER-2 molecular status in brain metastases from 77 NSCLC patients. Cancer Med. 2013;2(3):296–304. https://pubmed.ncbi.nlm.nih.gov/23930206/. doi: 10.1002/cam4.82 23930206 PMC3699841

[12] Ali S, Górska Z, Duchnowska R, Jassem J. Molecular Profiles of Brain Metastases: A Focus on Heterogeneity. Cancers (Basel). 2021 May 28;13(11):2645. doi: 10.3390/cancers13112645 ; PMCID: PMC8198739. https://www.ncbi.nlm.nih.gov/pmc/articles/PMC8198739/. 34071176 PMC8198739

[13] Bonert MZU, Maung R, El-Shinnawy I, Naqvi A, Finley C, et al. Pathologist workload, work distribution and significant absences or departures at a regional hospital laboratory. PLoS ONE 17(3): e0265905 2022. https://pubmed.ncbi.nlm.nih.gov/35333879/. doi: 10.1371/journal.pone.0265905 35333879 PMC8956155

[14] Bonert M, Naqvi A, Rahman M, Marshall JK, Xenodemetropoulos T, Arora P, et al. Stability of diagnostic rate in a cohort of 38,813 colorectal polyp specimens and implications for histomorphology and statistical process control. Sci Rep. 2021;11(1):16942. https://pubmed.ncbi.nlm.nih.gov/34417490/. doi: 10.1038/s41598-021-95862-2 34417490 PMC8379162

[15] Quail DF, Joyce JA. The Microenvironmental Landscape of Brain Tumors. Cancer Cell. 2017 Mar 13;31(3):326–341. doi: 10.1016/j.ccell.2017.02.009 ; PMCID: PMC5424263. https://www.ncbi.nlm.nih.gov/pmc/articles/PMC5424263/. 28292436 PMC5424263

[16] Zhang Q, Abdo R, Iosef C, Kaneko T, Cecchini M, Han VK, et al. The spatial transcriptomic landscape of non-small cell lung cancer brain metastasis. *Nat Commun* 13, 5983 (2022). doi: 10.1038/s41467-022-33365-y 36216799 PMC9551067

[17] Negi SK, Guda C. Global gene expression profiling of healthy human brain and its application in studying neurological disorders. *Sci Rep* 7, 897 (2017). https://www.nature.com/articles/s41598-017-00952-9. doi: 10.1038/s41598-017-00952-9 28420888 PMC5429860

[18] Osmani L, Askin F, Gabrielson E, Li QK. Current WHO guidelines and the critical role of immunohistochemical markers in the subclassification of non-small cell lung carcinoma (NSCLC): Moving from targeted therapy to immunotherapy. Semin Cancer Biol. 2018;52(Pt 1):103–9. https://pubmed.ncbi.nlm.nih.gov/29183778/. doi: 10.1016/j.semcancer.2017.11.019 29183778 PMC5970946

[19] Rekhtman N, Ang DC, Sima CS, Travis WD, Moreira AL. Immunohistochemical algorithm for differentiation of lung adenocarcinoma and squamous cell carcinoma based on large series of whole-tissue sections with validation in small specimens. Mod Pathol. 2011;24(10):1348–59. https://pubmed.ncbi.nlm.nih.gov/21623384/. doi: 10.1038/modpathol.2011.92 21623384

[20] Ao MH, Zhang H, Sakowski L, Sharma R, Illei PB, Gabrielson E, et al. The utility of a novel triple marker (combination of TTF1, napsin A, and p40) in the subclassification of non-small cell lung cancer. Hum Pathol. 2014;45(5):926–34. https://pubmed.ncbi.nlm.nih.gov/24746197/. doi: 10.1016/j.humpath.2014.01.005 24746197 PMC4178947

[21] Sayeda S, Naqvi A, Begum H, Juergens RA, Finley C, Coschi CH, et al. Prevalence of Thyroid Transcription Factor-1 (TTF-1)-Negative Small Cell Carcinoma and Napsin A Positivity in Small Cell Carcinoma in a Cross-Sectional Study of Lung Core Biopsies. Cureus. 2023;15(4):e37015. https://www.ncbi.nlm.nih.gov/pmc/articles/PMC10151064/. doi: 10.7759/cureus.37015 37139017 PMC10151064

[22] Travis WD, Brambilla E, Noguchi M, Nicholson AG, Geisinger K, Yatabe Y, et al. Diagnosis of lung cancer in small biopsies and cytology: implications of the 2011 International Association for the Study of Lung Cancer/American Thoracic Society/European Respiratory Society classification. Arch Pathol Lab Med. 2013;137(5):668–84. https://pubmed.ncbi.nlm.nih.gov/22970842/. doi: 10.5858/arpa.2012-0263-RA 22970842 PMC4509741

[23] Takano K, Kinoshita M, Takagaki M, Sakai M, Tateishi S, Achiha T, et al. Different spatial distributions of brain metastases from lung cancer by histological subtype and mutation status of epidermal growth factor receptor. Neuro Oncol. 2016;18(5):716–24. https://pubmed.ncbi.nlm.nih.gov/26519739/. doi: 10.1093/neuonc/nov266 26519739 PMC4827044

[24] Hammond ME, Hayes DF, Dowsett M, Allred DC, Hagerty KL, Badve S, et al. American Society of Clinical Oncology/College of American Pathologists guideline recommendations for immunohistochemical testing of estrogen and progesterone receptors in breast cancer (unabridged version). Arch Pathol Lab Med. 2010;134(7):e48–72. https://pubmed.ncbi.nlm.nih.gov/20586616. doi: 10.5858/134.7.e48 20586616

