# Supplementary material for: Neuroanatomical location of lung cancer brain metastases in 234 patients with a focus on cancer subtyping and biomarkers
Source: PLoS One. 2024 Nov 21;19(11):e0314205. doi: 10.1371/journal.pone.0314205 (PMC11581295; doi:10.1371/journal.pone.0314205)
Supplement: S1 File — (DOCX) [file pone.0314205.s001.docx]

# Data availability and Data File Description

The minimal anonymized data to reproduce the analysis herein is provided as a csv file in the supporting information for this manuscript. The included data file has 511 rows of data - one row for each patient.

The data columns are as follows:

clinical history (<0=discordant, 0=non-contributory, 1=contributory, 2=correct differential diagnosis)

primary site (LUNG = Lung, MELANOMA = Melanoma, BREAST = Breast, CRC = Colorectal, KIDNEY = Kidney, GI_NOS = Gastrointestinal Not Otherwise Specified, GYNE = Gynecologic, OTHER = urothelial, prostate, thyroid, germ cell tumour)

reported lung cancer subtype (NA = not applicable, ADENO = adenocarcinoma, NSmCC = non-small cell carcinoma, SmCC = small cell carcinoma, LCNEC = large cell neuroendocrine carcinoma, SqCC = squamous cell carcinoma)

IHC corrected lung cancer subtype (NA = not applicable, ADENO = adenocarcinoma, NSmCC-NOS = non-small cell carcinoma-not otherwise specified, SmCC = small cell carcinoma, LCNEC = large cell neuroendocrine carcinoma, SqCC = squamous cell carcinoma)

location (frontal (1=lesion present, 0=lesion absent), parietal (1=lesion present, 0=lesion absent), temporal (1=lesion present, 0=lesion absent), occipital (1=lesion present, 0=lesion absent), cerebellum (1=lesion present, 0=lesion absent), other location (1=lesion present, 0=lesion absent)), unique anonymous identifier (unique_anon_id)

TTF-1 corrected (1 = positive, -1 = negative, -99 = not available)

p63 corrected (1 = positive, -1 = negative, -99 = not available)

EGFR status (1 = positive, -1 = negative, -99 = not available)

ALK status by IHC (1 = positive, -1 = negative, -99 = not available)

PD-L1 status (2 = positive, 1 = low positive, -1 = negative, -99 = not available)

RAS status (1 = positive, -1 = negative, -99 = not available)

BRAF V600 status (1 = positive, -1 = negative, -99 = not available)

ROS-1 status (1 = positive, -1 = negative, -99 = not available)

ALK status by FISH (1 = positive, -1 = negative, -99 = not available)

Year (11, 12, 13... 20)

Anonymous identifier

Cases were sorted by anonymous identifier.
