# Supplementary figures and images for: Neuroanatomical location of lung cancer brain metastases in 234 patients with a focus on cancer subtyping and biomarkers
Source: PLoS One. 2024 Nov 21;19(11):e0314205. doi: 10.1371/journal.pone.0314205 (PMC11581295; doi:10.1371/journal.pone.0314205)

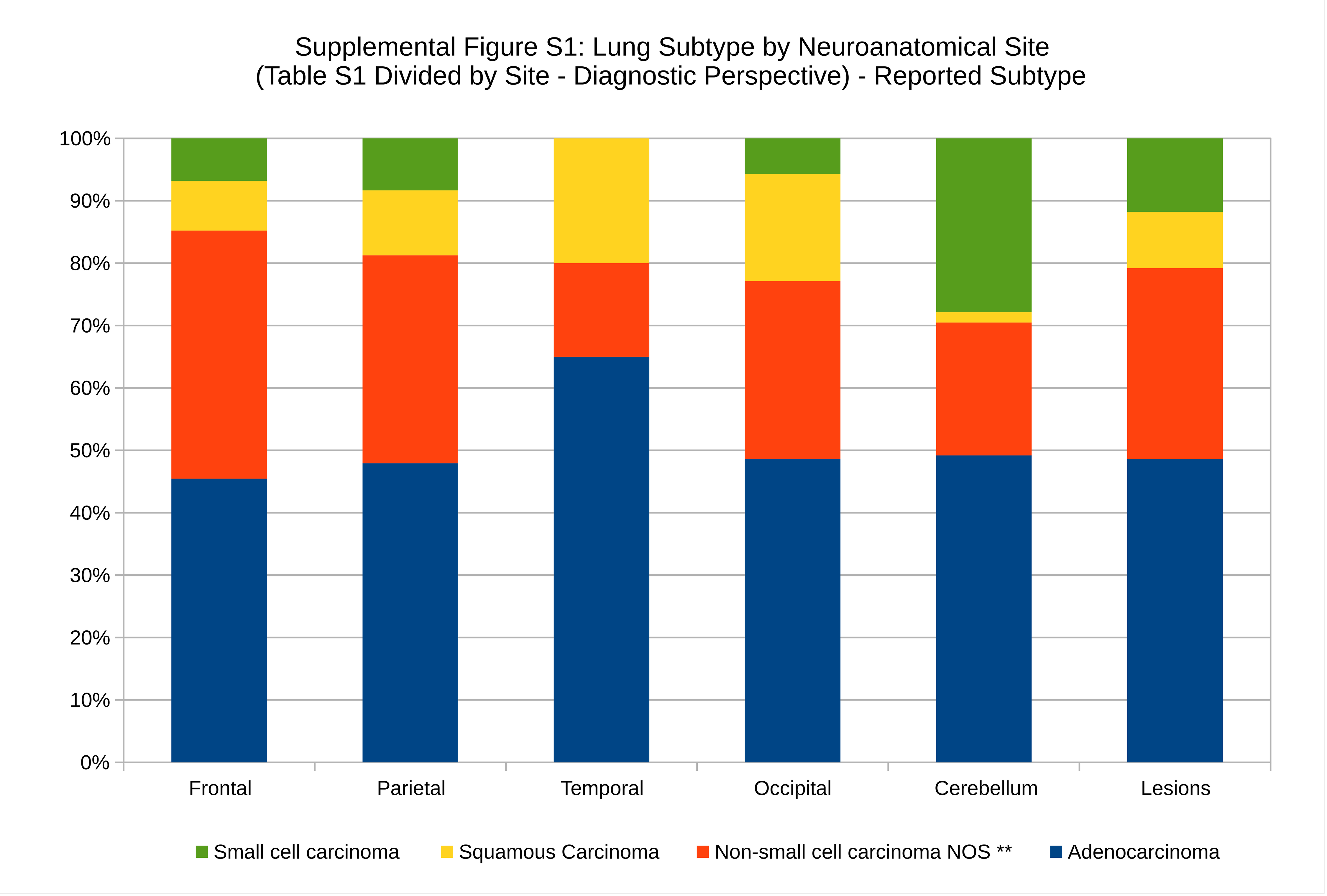

Supplement: S1 Fig — (TIF) [file pone.0314205.s004.tif]

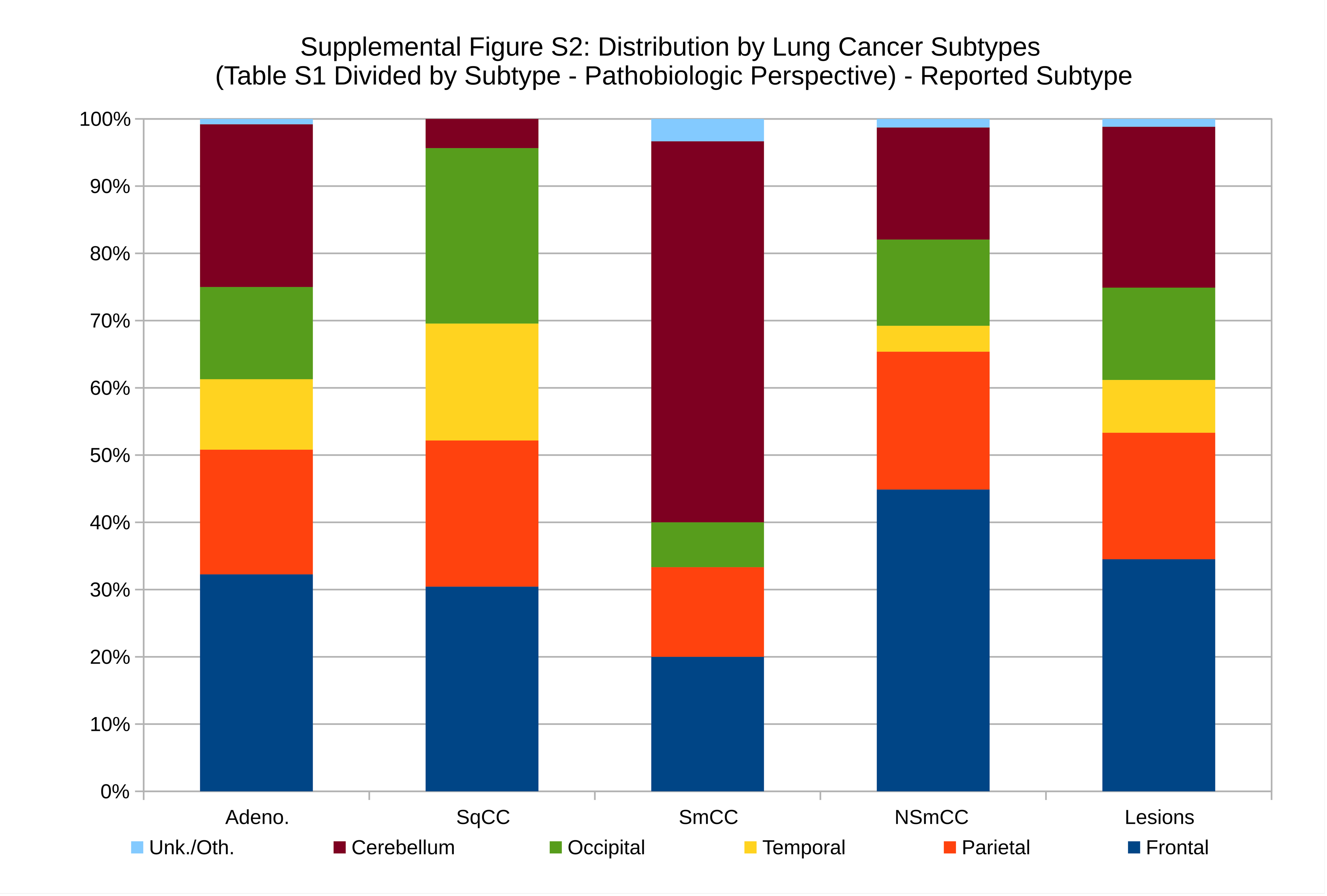

Supplement: S2 Fig — (TIF) [file pone.0314205.s005.tif]

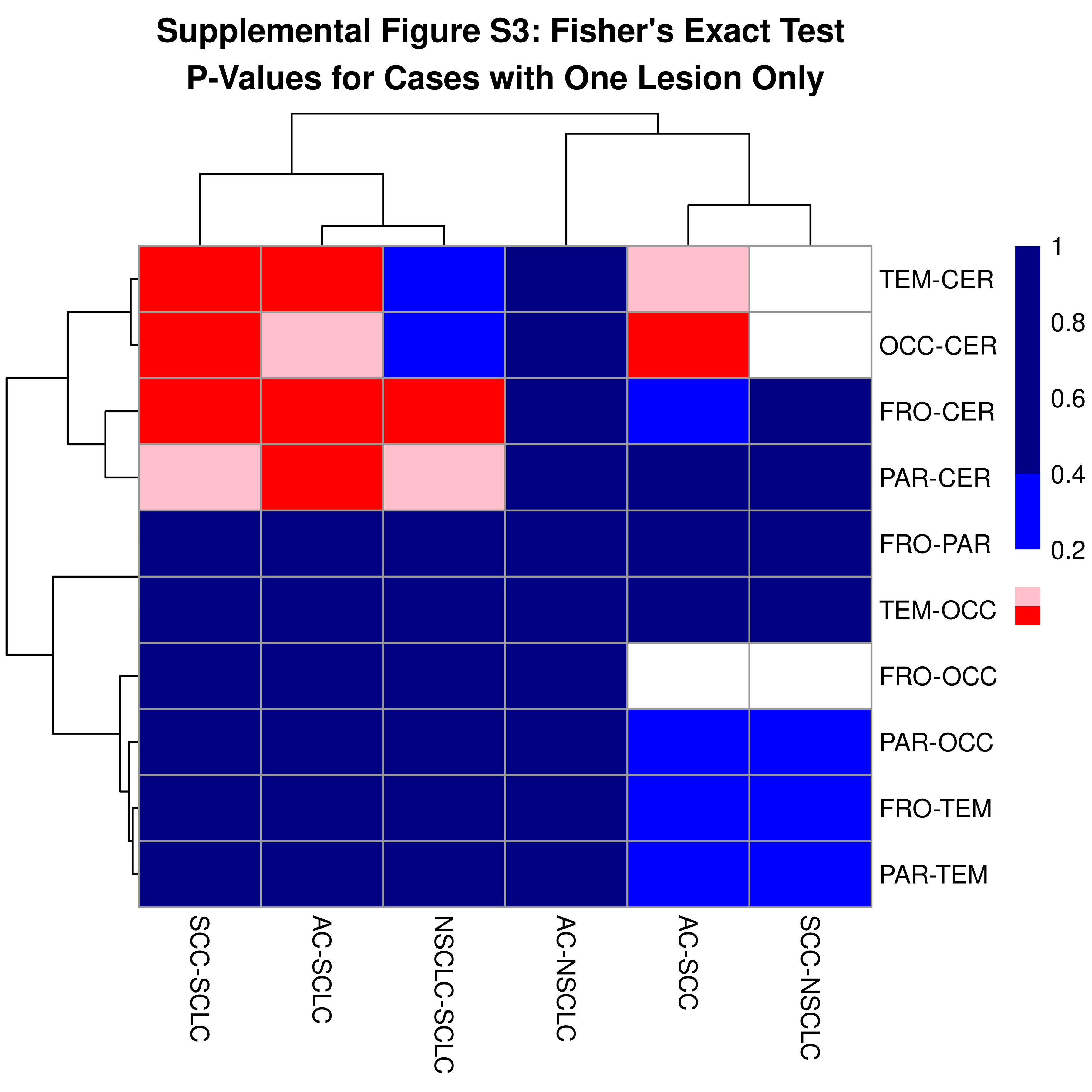

Supplement: S3 Fig — (TIF) [file pone.0314205.s006.tif]

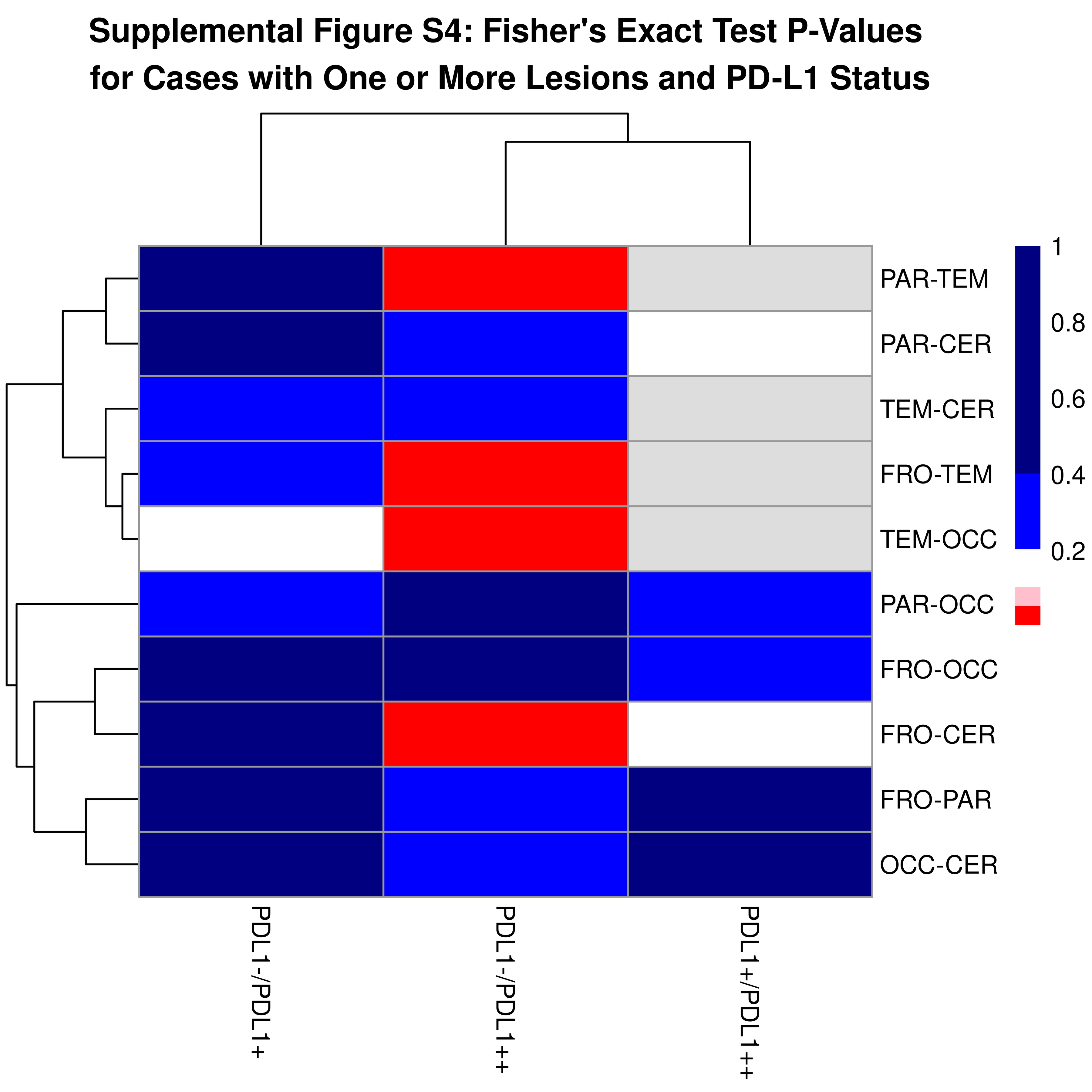

Supplement: S4 Fig — (TIF) [file pone.0314205.s007.tif]
